# Environmental exposures and epigenetic alterations in common chronic diseases: insights and challenges

**DOI:** 10.1093/eep/dvag014

**Published:** 2026-04-17

**Authors:** Cathrine Hoyo, Chantel L Martin, Terrence Allen, David Skaar, Susan K Murphy

**Affiliations:** Department of Biological Sciences, North Carolina State University, Raleigh, NC 27606, United States; Center for Human Health and the Environment, North Carolina State University, Raleigh, NC 27606, United States; Department of Epidemiology, University of North Carolina at Chapel Hill, Chapel Hill, NC 27599, United States; Department of Anesthesiology, Duke University School of Medicine, Durham, NC 27710, United States; Department of Biological Sciences, North Carolina State University, Raleigh, NC 27606, United States; Center for Human Health and the Environment, North Carolina State University, Raleigh, NC 27606, United States; Department of Obstetrics and Gynecology, Duke University School of Medicine, Durham, NC 27708, United States

**Keywords:** environmental epigenetics, developmental windows of susceptibility, DNA methylation, imprinting control regions, epidemiology

## Abstract

Exposure to environmental factors including contaminants and social conditions is implicated in a substantial proportion of common non-communicable diseases, and data from model systems repeatedly demonstrate that the process from environmental contributions to common chronic disease risk is mediated through maladaptive epigenetic responses. The field of environmental epigenetics leverages multiple disciplines to advance our understanding of environmental impacts on epigenomic processes to enhance etiologic investigation, guide biomarker discovery, and identify mechanisms of action that ultimately lead to behavioral and or therapeutic interventions. This article discusses examples of emerging research on the links between three common life course exposures linked to common non-communicable diseases, and their associated epigenetic modifications, with a major focus on DNA methylation—the most studied in humans. It also outlines current challenges when interpreting the accumulating body of knowledge, including the lack of consensus on regions reported to be targeted by these environmental exposures. Finally, given that the strongest predictors of epigenetic states are age and cell/tissue type, strategies to build novel platforms using existing technologies to surmount some of these challenges are discussed. Together, these advances in environmental epigenetics are paving the way for groundbreaking developments toward improved precision in developing prevention and intervention strategies to reduce common non-communicable disease morbidity and mortality.

## Introduction

Environmental exposures that include physical factors such as extreme temperatures, contaminants, and social stressors like housing quality are estimated to account for up to 90% of the risk for common non-communicable diseases [1]. These diseases, including cardiovascular and cerebrovascular disease, cancers, metabolic, and neurological diseases, account for 75%–80% of all deaths in the United States and globally [1, 2]. While occupational epidemiologic and experimental toxicologic studies have demonstrated the deleterious effects of high level or prolonged singular environmental exposures to human health, the health effects of multiple chronic low-dose exposures experienced broadly through industrialization, fossil-fuel combustion, and chemical-intensive agriculture remain inconsistent and difficult to interpret. These uncertainties impede causal inference, complicate prevention efforts, and hinder the development of effective interventions and policies. Consequently, the field of environmental epigenetics, i.e. the study of how environmental exposures induce changes in gene expression without altering the DNA sequence, has expended considerable effort to improve our understanding molecular pathways through which the environment contributes to these common non-communicable diseases [3, 4].

Molecular changes that transiently or stably alter organ structure and function contribute to common non-communicable diseases largely through epigenetic mechanisms, such as DNA methylation, histone modifications, and non-coding RNA molecules. Although human data remain limited, model systems data show that some epigenetic alterations can be transmitted across generations—*inter-generationally* when the F1 generation and its germ cells are directly exposed in utero, and *trans-generationally* when exposure-related changes appear in unexposed F2 or F3 descendants [5, 6]. A major motivation for studying these mechanisms is their potential to serve as “archives” that reflect prior environmental exposures and, in turn, biomarkers that help identify individuals at elevated risk for chronic disease long before symptoms arise [7]. DNA methylation is particularly promising in this regard: as a covalent and mitotically stable modification, it can “archive” historical or cumulative exposures, especially when exposures are intermittent, difficult to measure, or poorly recalled. Unlike questionnaires, short–half-life biomarkers, or environmental monitoring, many methylation signatures integrate exposure effects over long periods, while others may function as biosensors of contemporary exposures or subclinical disease, offering opportunities for earlier detection and intervention [8]. Moreover, because age-related methylation changes influence genomic stability and gene expression, these markers may also help pinpoint developmental windows during which environmental exposures exert their greatest effects and tailoring interventions accordingly [7]. Although epigenetic regulation involves multiple interacting layers, including histone modifications and chromatin accessibility, this review focuses on DNA methylation because of its stability, functional relevance, and practical utility for biomarker development.

DNA methylation is especially valuable in human studies because it can be reliably measured from nearly any biospecimen, across the life course, regardless of preservation, or extraction protocols, using both targeted and genome-wide approaches. The covalent 5-methylcytosine bond and the inherent chemical stability of DNA confer persistence, enabling methylation marks to function as retrospective indicators of past environmental exposures that inform both disease risk and underlying mechanisms based on the functional role of affected genomic regions. Genome-scale DNA methylation profiling is achieved using several platforms, including whole genome bisulfite sequencing (WGBS), reduced representation bisulfite sequencing (RRBS), methylated DNA immunoprecipitation (MeDIP), or array-based technologies, such as the Beadchip arrays, with genomic coverage ranging from 3% to 5% for arrays to nearly complete coverage for WGBS. Although the functional consequences of methylation changes in gene bodies and intergenic regions are often less well defined, promoter-associated methylation—well captured by existing array platforms—has established regulatory effects on gene expression. Beyond locus-specific analyses, DNA methylation data have also enabled the development of epigenetic clocks, which use data-driven algorithms to estimate biological age from methylation patterns across hundreds to thousands of CpG sites and serve as surrogate markers of aging-related morbidity and mortality risk, with accelerated epigenetic aging defined as DNA methylation age exceeding chronological age [9].

While some of this effort is underway, realizing the potential of environmental epigenetics urgently requires substantial advances in computational capacity and data sharing to rapidly build a deployable compendium of epigenetic marks that are responsive to major classes of environmental exposures for mechanistic interrogation. The objective of this review is three-fold. First, it synthesizes findings on epigenetic associations with three common exposure domains during select developmental windows—prenatal environmental toxic metals and pharmaceutical exposures and social stressors at the individual and area levels across the life course where fetal epigenetic data remain limited—selected because they serve as exemplars for broader classes of chemical and non-chemical stressors. Second, we outline key methodological and practical challenges to interpreting the accumulating evidence linking these environmental exposures to epigenetic alterations. Third, we discuss strategies, some leveraging existing technologies, to build scalable and reproducible platforms, within developmental windows, for overcoming these challenges.

## Epigenetic response to environmental stressors and health effects

### Toxic metals exposure

#### Toxic metals and common chronic diseases

Lead and cadmium are common, naturally occurring elements found on the earth’s surface, with properties that have led to their mining, refining, and use in a wide range of industrial applications [10]. The long-running release of these elements into the environment has resulted in widespread contamination of our water and food supply. Lead exposure has been associated with a wide range of outcomes throughout the life course, including neurological [11] and cardiovascular [12] diseases in adults. Data accumulated in the last 30 years also support that *in utero* exposure to these elements is associated with neurodevelopmental [13–15] and cardiometabolic dysfunction in children [16]. Similarly, *in utero* cadmium exposure, which sometimes co-occurs with lead exposure [17, 18], is associated with a wide range of metabolic outcomes in children and adults, affecting the lung, liver, prostate, kidney, bladder, stomach, and pancreas (reviewed by [19]). *In utero* cadmium exposure is associated with poor birth outcomes that are established risk factors for metabolic impairment later in life, including lower birth weight, shorter length, and smaller head circumference [20–27]. A recent review noted that many of these health effects were consistent in model systems for both lead and cadmium but that only approximately 50% of published human studies supported similar findings [28].

#### Toxic metals and epigenetics

Exposure to toxic metals can exert long-term—and in some cases intergenerational or transgenerational—effects on health, which has prompted intensive investigation into associated epigenetic marks, particularly DNA methylation. A subset of sequence regions of particular interest for studies of exposure effects in early development are differentially methylated regions (DMRs) with parent-of-origin specific allelic methylation, established shortly after fertilization, maintained through differentiation, and generally stable across the life span. A well-studied example is the Delta-like 1/Maternally Expressed Gene 3 (*DLK1/MEG3*) imprinted domain on chromosome 14q32.2 that regulates the expression of growth effectors *MEG3* and *DLK1*, in addition to an entire gene cluster that includes *MEG8, RTL1, DIO3*, and large numbers of small nucleolar RNAs (snoRNAs) and microRNAs. The *MEG3*-IG DMR is located 13 kb upstream of the *MEG3* transcription start site; in contrast, the *MEG3* DMR is intragenic and overlaps with the *MEG3* promoter. While the *MEG3*-IG DMR is the main imprinting control region (ICR) that functions to affect proper allelic expression of the genes and RNAs in this domain, the *MEG3* DMR maintains the active (unmethylated) status at the IG DMR on the maternal allele and functions in somatic tissues, allowing for expression of downstream genes [29–31]. Hypermethylation of the *MEG3* DMR has been associated with multiple forms of metabolic-associated diseases including multiple cancers [32]. Notably, after accounting for co-occurring toxic metal(oid)s, lead and arsenic, and other prenatal factors previously shown to alter CpG methylation at birth, *in utero* cadmium exposure was associated with hypermethylation of the *MEG3* DMR [18], findings corroborated by geospatial analysis demonstrating that hypermethylation of the *MEG3* DMR coincided with elevated cadmium exposure previously reported [33, 34]. These data are consistent with the responsiveness of this DMR to environmental cadmium.

Beyond these targeted analyses, data using genome scale approaches has been recently reviewed by Elkin [11] and Dutta [10]. For example, in an investigation using WGBS in maternal and umbilical cord blood mixed leukocytes, Cowley et al. [35] identified 641 DMRs (with > 10% methylation difference) in newborns of women with elevated early-gestation cadmium levels; 1940 DMRs were detected in maternal leukocytes, with enrichment in metabolic, liver, cardiovascular, and obesity-related pathways. Remarkably, 98 DMRs mapped to genes shared by mothers and newborns, consistent with potential intergenerational epigenetic inheritance. Multiple regions targeted by toxic metals have also been identified using the HumanMethylation450K/EPIC array although few are replicable. For example, in Bangladeshi mother-child pairs, 10 DMRs identified in cord blood mixed leukocytes replicated in peripheral blood mononuclear cell-derived DNA at 9 years of age [36]. Among Koreans, *in utero* differential methylation of probe cg21010642 mediated the association between cadmium exposure and shorter gestational age [37], and cigarette smoking—a source of cadmium—was significantly associated with hypomethylation of probe cg05537752, a site annotated to the *ATP9A* gene [38], and previously reported by others [38–40].

Of studies evaluating *in utero* lead exposure and DNA methylation, higher concentrations of lead during the first trimester were associated with DNA methylation differences at three CpG sites among Mexican women and their offspring, yet no significant associations were found with elevated second trimester levels, and only two CpG sites were associated with third trimester levels [41]. These associations have been confirmed by others, together with the lack of consensus on the timing with some reporting stronger and sex-specific effects in the first trimester and others in later trimesters [42, 43]. In U.S. children, nine CpG sites associated with first-trimester lead exposure persisted into mid-childhood [44].

Thus, collectively, human data largely support that prenatal toxic metals such as lead and cadmium are associated with DNA methylation differences in the offspring, with associations varying by sex or developmental window in which exposure occurs, with some associations persisting into childhood. However, except for a small number of CpG sites, the majority of methylation marks associated with exposure to these two common toxic metals do not appear to be replicable across studies.

### Social environmental stressors

#### Social stressors and epigenetics

Individual social stressors during various developmental windows, including the prenatal and early postnatal periods, can potentially increase the risk of adverse metabolic and neurodevelopmental outcomes through epigenetic perturbations. Early indications that well-measured social stressors were associated with epigenetic perturbations in humans came from statistical models using targeted analysis to identify DNA methylation marks associated with maternal education [45]. This analysis culminated in a recent publication identifying 573 CpG dinucleotides at birth associated with maternal education among *n* = 9881 offspring across six continents [45]. Some of these differences remained detectable when measured again in childhood (*n* = 2017, 1 site) and adolescence (*n* = 2740, four sites). These CpG sites mapped to or near genes enriched for maternal folate, vitamin-B_12_ concentrations, maternal smoking, and maternal obesity. Others measured socioeconomic status directly using the British system of social class. In mixed leukocyte-derived DNA, a small study used Infinium 450K methylation arrays, of males from socioeconomic position extremes in both early childhood and mid-adulthood included in the 1958 British Birth Cohort Study. The investigators reported 9112 CpG sites mapping to or near 6176 genes with 1252 of these genes associated with childhood socioeconomic position and 545 associated with adulthood socioeconomic position at 45 years of age [46]. CpG sites associated with childhood socioeconomic position were in promoter regions of genes enriched in key cell signaling pathways, such as MAPK signaling, BDNF receptor signaling, and hormone receptor signaling among others. Since these studies, researchers have hypothesized that these individual-level social stressors co-occur with area-level social stressors, such as neighborhood stressors, to influence DNA methylation [47].

#### Neighborhood social environment and epigenetics

With the realization that individual socioeconomic positions occur within the neighborhood context, neighborhood social stressors began to be measured using publicly available administrative data (i.e. information from the U.S. Census Bureau, geographic information systems, neighborhood audits and evaluations, and residential surveys) across multiple geographic levels, including county, census tract, census block groups, and residential buffers. Research into neighborhood social stressors has consistently shown that individuals residing in neighborhoods with higher exposure to social stressors (i.e. segregation, socioeconomic disadvantage, violence) are more likely to suffer higher burdens of adverse health outcomes [48, 49]. Although studies of epigenetic mechanisms linking neighborhood social stressors to health have increased over the past decade, this body of research is still emerging, yet the evidence is compelling.

Some of the earliest research examined DNA methylation of the regulatory regions of imprinted genes and stress–response- and inflammation-related pathways. One of the early studies identified an association between prenatal neighborhood disadvantage (measured as six census tract-level socioeconomic variables) and increased methylation of the *MEG3* DMR in offspring cord blood leukocytes [34]. The *MEG3* gene, which produces a long non-coding RNA has been implicated in many cancers. Since this study, researchers have conducted analyses of neighborhood social environmental stressors on DNA methylation at other genomic regions [50–52]. For example, DeLano et al. [50] examined the association between prenatal neighborhood deprivation and DNA methylation in three stress-related genes (*NR3C1, SLC6A4*, and *SCG5*) measured in buccal cell-derived DNA collected from infants at 4 weeks postpartum. They found that increased neighborhood deprivation during pregnancy was associated with increased methylation across multiple CpG sites in *SLC6A4*. Interestingly, results from an adult cohort found increased methylation in the *SLC6A4* gene in relation to neighborhood socioeconomic disadvantage (measured as 16 census tract-level variables) and self-reported adverse social environment (captured using self-administered questions on aesthetic quality, safety, and social cohesion), along with several additional stress- and inflammation-related genes [52]. One of the few prospective studies of childhood neighborhood socioeconomic disadvantage and DNA methylation at 18 years of age also identified associations between neighborhood socioeconomic disadvantage and differential DNA methylation in genes related to inflammation [51]. These studies illustrate that targeted gene approaches can shed critical insights and uncover key mechanisms for further epigenetic and molecular interrogation.

More recently, DNA methylation data across multiple tissues and cell types have been used to develop epigenetic clocks as summary estimators of cellular aging in population health research [53], primarily to evaluate the effect of social stressors (*and other environmental exposures*) on epigenetic aging. For example, multiple studies have reported associations between neighborhood social environments, including socioeconomic disadvantage, disorder, poverty, residential segregation, and low social cohesion, and accelerated epigenetic aging: these findings are consistent using several epigenetic clocks from birth through adulthood [54–63]. Importantly, accelerated epigenetic aging has itself been linked to a range of aging-related health outcomes and increased mortality risk [64, 65]. Together, these findings suggest that socially patterned environmental exposures may become biologically embedded through accelerated epigenetic aging. This underscores the potential of DNA methylation–based aging biomarkers to elucidate biological pathways linking social conditions to long-term health, while also highlighting the need for further research to establish causal relationships.

Thus far, only a small number of studies has evaluated the effect of neighborhood social factors on DNA methylation using genome-scale approaches [51, 62, 66–69]. Multiple studies have sought to identify potential epigenetic targets linking the neighborhood social environment to breast cancer disparities, identifying several differential methylated CpG sites in relation to various neighborhood social factors, such as contemporary redlining and neighborhood deprivation [62, 67, 68]. A recent study identified DMRs in offspring cord blood associated with gestational exposure to neighborhood crime [66]. A study of prefrontal cortex brain tissue from 159 donors found an association between neighborhood deprivation and differential methylation at one CpG site in the gene *PLXNC1* that was significantly modified by the *APOEε*40 allele, a well-known risk factor for Alzheimer’s disease [69]. Finally, Reuben et al. (2020) found that children raised in neighborhoods with greater disadvantage had differential DNA methylation in mixed blood leukocytes at 18 years of age at six CpG sites across four genes (*CNTNAP2, CYP1A1, AHRR*, and *OR4C13*) [51]. While these findings are important to the discovery of novel pathways, replication studies to corroborate identified CpG sites have either not been conducted or findings fail to replicate.

### Exposure to pharmaceuticals in early life

#### Prenatal exposure to general anesthesia

Prenatal anesthesia and analgesia exposures have recently been in the spotlight due to concerns about their long-term effects on neurodevelopmental outcomes in the offspring [70, 71]. Up to 1% of women require surgery during pregnancy, usually for emergency procedures such as appendicitis, cholecystitis, and adnexal masses [72], with 42%, 35%, and 23% of surgeries reported to occur during the 1st, 2nd, and 3rd trimesters, respectively [72]. While data from animal and a few human studies have raised concerns about the effects of general anesthesia on neurodevelopment, leading to the United States Food and Drug Administration issuing warnings in 2016 [70, 71], evidence demonstrating deleterious effects of general anesthesia on the human fetus is inconsistent [70, 71]. For instance, a recent study identified associations between general anesthesia exposure in pregnant women undergoing non-obstetric surgery in the 1st, 2nd, or 3rd trimester of pregnancy and an increased risk of disruptive and or internalizing behavioral disorders in index offspring [71]. The risk for these neurobehavioral outcomes was higher for children exposed during the 2nd or 3rd trimester when compared to those exposed in the 1st trimester. The effects of general anesthesia on neurodevelopment have been extensively reviewed, but this has been to primarily address concerns about the neurodevelopmental effects of anesthesia in neonates and very young children [73–75].

#### General anesthesia and epigenetics

Existing data suggest that exposure to general anesthetic agents can adversely affect brain development, synaptic plasticity, and neuronal function [73–75]. These associations are plausible because volatile anesthetic agents are low molecular weight, lipophilic molecules that undergo significant placental transfer to the fetus, and because developmental synaptogenesis continues for years postnatally, extending the window of vulnerability for the developing brain [74]. Beyond acute neurotoxicity to index offspring, these exposures can lead to chronic neuropathological changes that may be transmitted intergenerationally [75]. In search for mediating epigenetic mechanisms, preclinical models have identified dysregulation of both DNA methylation and histone acetylation in response to repeated exposure with sevoflurane, a commonly used volatile general anesthetic agent: exposure resulted in significant upregulation of the de novo DNA methyltransferases, Dnmt3a and Dnmt3b, at the mRNA and protein levels in rodent pups [76]. These exposures were associated with loci-specific increased DNA methylation in the postsynaptic density protein 95 (*Psd95*) and synaptophysin (*Syp*) genes, which regulate both synaptic plasticity and function [75, 76]. Exposure to a triple cocktail of anesthetic agents in rodents led to a significant reduction in brain-derived neurotropic factor (*BDNF*) and cellular Finkel-Biskis-Jinkins murine sarcoma virus osteosarcoma oncogene *(c-fos*) mRNA and protein expression (c-fos genes), likely mediated via anesthesia-induced hypoacetylation of their promoters through reduced CREB binding protein histone acetyltransferase (CBP-HAT) activity in the hippocampus [77]. Both *Bdnf* and *c-fos* are critical genes in neuronal development [77].

Several lines of evidence also indicate that early exposure to general anesthetic may also be a powerful epigenetic modulator with intergenerational effects on neuronal maturation, apoptosis, circuitry formation, and neurobehavioral and cognitive development [78, 79]. For example, exposure to general anesthesia in male rodent pups on postnatal day (PND) 5 resulted in potassium chloride cotransporter 2 (*Kcc2)* gene hypermethylation in sperm. This epigenetic modification was also evident in the next generation of male offspring with no direct exposure to general anesthesia [78]. Furthermore, these offspring demonstrated deficiencies in hippocampal learning and memory, although it is unclear if these results are directly attributable to *Kcc2* hypermethylation [78]. Moreover, female neonatal pups who were exposed to general anesthesia exhibited significant hypomethylation of genes encoding transcription factors junb proto-oncogene (*Junb*) and activity related cytoskeleton associated protein (*Arc*) in the subiculum. This effect also persisted in the next generation of juvenile offspring despite these offspring never having direct exposure to anesthesia themselves [79].

#### Epidural anesthesia and opioids

Another underappreciated exposure arises from the widespread use of labor epidural analgesia, which is used by approximately 70% of parturients in the US, and is the current gold standard for labor pain management, with global use continuing to rise [80, 81]. This technique involves administration of low concentrations of local anesthetics (e.g. bupivacaine or ropivacaine) combined with a lipophilic opioid such as fentanyl into the lumbar epidural space, with higher anesthetic concentrations used when conversion to Cesarean delivery is required. Compared with systemic opioids or no analgesia, epidural anesthesia is associated with reduced severe maternal morbidity, lower pain scores, greater maternal satisfaction, and decreased need for additional analgesia [80]. However, data on potential long-term effects in offspring remain limited.

#### Epidural anesthesia, opioids, and epigenetics

A recent study reported an association between labor epidural anesthesia exposure and autism in the offspring [82], although subsequent studies have failed to replicate this association [83–85]. In contrast, a protective effect for childhood asthma was identified in association with longer duration of exposure to labor epidural analgesia—with associations mediated by epigenetic shifts reported [86, 87]. In that report, 20 differentially methylated CpG sites mapping to 21 genes measured in umbilical cord blood-derived DNA mediated this association [87]. These genes were enriched for immune-mediated and inflammatory pathways with regulatory networks related to the MHC class I and NFKB complexes. It is still unclear if these epigenetic effects are an indirect result of attenuation of the maternal and fetal physiological stress response to labor pain, or due to direct exposure to local anesthetics and opioids administered maternally into the epidural space for labor analgesia. Bupivacaine, one of the most common local anesthetics administered for labor analgesia, and its metabolite, 2,6 pipecolyxylidine (PPX), remain detectable in neonatal urine up to 3 days following administration [88, 89]. About 90% of epidurally administered fentanyl, a μ-opioid receptor agonist, also undergoes rapid transplacental transfer to the fetus [90]. Mechanistically, emerging data among adults now suggests local anesthetics may act as non-nucleoside DNA demethylating agents indirectly through effects on miRNA expression impact histone acetylation promoting anti-inflammatory and antitumor immune effects [91]. Opioids appear to increase global DNA methylation levels [92]. In a mouse model, fentanyl administered to young mice of both sexes induced autism-like behavior partly mediated by hypermethylation of the glutamate receptor gene *Grin2b* in the anterior cingulate cortex of the brain [93].

Beyond neurocognitive, respiratory, and neurobehavioral effects, emerging data suggest prenatal opioid exposure may also lead to aberrant growth patterns and dysregulated feeding behavior in neonates, placing them at risk for developing metabolic dysfunction, including obesity, in later life. The μ-opioid receptor (MOR, encoded by *OPRM1*) plays a pivotal role in controlling the reward properties of natural stimuli such as palatable foods that, in addition to opiates, hedonically drive the feeding process [94, 95]. Activation of MOR in the ventral striatum leads animals to selectively seek out high fat foods, while stimulation of MOR in the cortex increases the consumption of a high carbohydrate diet [96]. Furthermore, obesity modulates the opioid system with MOR upregulation in offspring of mothers who consumed a high fat diet [97]. Dams who consumed a high fat diet produced male offspring with altered expression of the MOR, hyperphagia, and preference for high fat diets [98]. Evidence for the potential role of epigenetic deregulation in this interplay between obesity, dysregulated feeding behavior, prenatal opioid exposure, and opioid receptor function was corroborated by reduced DNA methylation in the promoter regions of *OPRM1* and the dopamine reuptake transporter, *DAT*, in the mouse brain in gestational and pre-pregnancy obesity model systems—which were reversed by MOR antagonists or methyl donor supplementation [99, 100]. While these adverse metabolic effects have been studied in the setting of chronic opioid use encountered with opioid use disorders (OUD) in pregnancy, it remains unclear whether prenatal exposure to intravenous and epidural opioids in labor may have a similar effect beyond issues with breastfeeding that have been previously described [101–104]. Additionally, aside from OUDs, treatment for OUDs, and labor analgesia, opioid use in pregnancy is common, with up to 22% of Medicare beneficiaries and 14% of privately insured beneficiaries receiving at least one opioid prescription during pregnancy [105].

## Challenges in interpreting existing epigenetic data

Despite their potential to function as archives of past exposure—thereby improving exposure assessment by capturing historical or cumulative exposures, particularly when exposures are intermittent or poorly recalled—substantial challenges remain before this potential can be fully realized. In this section, we discuss existing hurdles to the implementation of DNA methylation as effective and accurate indicators of historical exposures or biosensors. These challenges include technological limitations in measurement and analysis, the plasticity and tissue specificity of many DNA methylation marks in response to environmental exposures, limited population diversity in existing studies, and incomplete understanding of the underlying biological mechanisms.

### Technological challenges and associated costs

The ability to detect DNA methylation differences has advanced markedly, from targeted locus-specific analysis using restriction digestion to agnostic whole genome approaches in under two decades. Although whole-genome bisulfite sequencing remains the gold standard, for cost efficiency, current utilitarian, agnostic approaches commonly rely on array-based technologies, such as the Illumina Infinium MethylationEPIC v2.0 BeadChip, which now measures CpG methylation at approximately 930 000 sites across gene promoters and gene bodies and captures ∼96% of CpG islands (including shores and shelves) annotated in the University of California, Santa Cruz Genome Browser. Data generated using this technology and its prior iterations (e.g. the 27k and 450k BeadChips) has been informative, is reproducible, and has facilitated rapid measurements of large numbers of specimens. For studies conducted among adults, this methylation array technology has made possible rapid measurement of CpG methylation using DNA, previously archived from epidemiologic and clinical studies of common non-communicable diseases, including cardiovascular disease, generating new hypotheses. In children’s studies, the high reproducibility afforded by this array technology has enabled the formation of consortia, such as the international Pregnancy and Childhood Epigenetics (PACE) Consortium [106], comprising more than 30 birth cohort studies across five continents with tens of thousands of umbilical cord blood DNA samples measured. In PACE, CpG methylation array data are being statistically evaluated in relation to environmental exposures, outcomes and as mediators where possible. These consortia arrangements maximize statistical power and simultaneously facilitate assessment of the effects of CpG methylation in populations with different confounding structures, globally. In addition to high power for robust statistical assessment, replication among these diverse populations can be achieved with ease—an important criterion for causal inference. While more diversity is still needed to ensure inclusion of ethnic minorities and rural residents who rarely participate in studies, one striking example from the PACE consortium is the discovery of the hypomethylation of the CpG site (Infinium probe ID cg05575921) in the aryl hydrocarbon receptor gene (*AHRR*) in association with maternal cigarette smoking in 13 cohorts comprising 6685 cord blood-derived DNA samples [106]. This finding has been replicated in children and adults [107–110] and is being proposed for use as a biomarker of cigarette smoking for use by insurance companies.

Nonetheless, while agnostically screening across the genome with high reproducibility is a strength, physical limitations constrain array capacity. There are ∼28 million CpG sites in the human genome; the EPIC BeadChip provides only ∼3.3% coverage of all possible sites. Its design was intentional and targeted to CpG islands, shores and shelves, CpG sites that were known to exhibit variable methylation from prior studies, those known to be disease-associated (e.g. cancer-related), and those in known or putative regulatory regions, among others. Another genome-scale tool, methylated DNA immunoprecipitation (meDIP), covers roughly 10%–30% of the genome but is dependent on antibody precipitation of methylated cytosine and is thus biased toward capture of CG-rich regions. RRBS is another genome-scale approach, covering approximately 15% of CpG sites due to its use of methylation insensitive restriction digestion targeting CG-containing recognition sites to generate DNA fragments that then undergo bisulfite conversion and sequencing. RRBS is also biased toward CG-rich regions of the genome but allows for single nucleotide resolution of DNA methylation within the sequenced regions. While these methods are useful and can be highly informative, many known epigenetic regulatory regions are not in immediate proximity to genes (≥tens of kb) or are located in areas of low CG content.

Whole genome methylation sequencing, with conversion of unmethylated cytosines to uracil by either bisulfite or enzymatic processes, or long-read Nanopore sequencing, which directly detects methyl-cytosine, provides the most thorough methods for measuring CpG methylation. These methods can quantify methylation at single-base resolution for the entire genome. Nanopore sequencing can also improve coverage in repetitive regions of DNA, where regulatory methylation often occurs, as compared to chemical or enzymatic methods. This improvement comes from both the preservation of all four DNA bases, and much longer read length (kilobases vs. 150–300 bp), which allow for unambiguous genomic assignments in regions where short, three-base reads have no unique alignment, causing the loss of up to 20% of genome sequence. Moreover, the newer Illumina NovaSeq XP Plus platform can generate 52 billion single reads per dual flow-cell run—enabling highly multiplexed sequencing of large sample sets. Combined with enrichment methods such as Twist BioSciences capture (San Francisco, CA) or methyl-CpG binding domain sequencing (MBD-seq), which specifically enrich methylated CpG sites, these technologies facilitate scalable methylation profiling across many samples. The coverage achieved by these platforms, their sequence read depth, and base call accuracy, combined with their increasing cost-effectiveness, have resulted in these combined whole genome approaches becoming the gold standard for detailed screening. The higher density coverage of these whole genome methods captures intergenic regions with low CpG density. There are known important regulatory regions located in such CpG- sparse regions, with still more having provisionally identified regulatory elements, but these regions are poorly covered by arrays, due to the targeted design, as well as the difficulty of assaying repetitive regions with the short probes sequences of arrays. Additionally, even where arrays target a region of interest, capacity limitations and sequence requirements restrict the number of CpG sites measurable, while whole genome sequencing has no such restrictions.

Finally, determining allele-specific patterns and parent-of-origin methylation is also challenging. Only long-read sequencing can provide the physical connection between an ICR and informative (heterozygous) sequence polymorphisms (“phasing”), thus distinguishing alleles and assigning parental origins, given one known parental genotype. Several studies have reported using phased sequencing to identify likely ICRs and their parental origins, particularly leveraging long-read platforms such as Oxford Nanopore (Oxford, UK) and PacBio (Menlo Park, CA) HiFi, which provide the read lengths necessary for haplotype resolution in these analyses, often in combination with complementary phasing methods. Although when combined, these strategies can reduce costs, it remains a major bottleneck (∼$1000 per sample) especially when combined with the storage computational challenges, which include not only computational space given the tera-bases of data delivered, but also the needed deployment of non-standard bioinformatics and statistical pipelines.

### Plasticity/Stability of DNA methylation marks

The plasticity of DNA methylation marks in response to environmental exposures during the life course in combination with their covalent nature and somatic heritability can create a stable record of exposure history [7]. Moreover, the specific developmental window or timing during the life course when an exposure occurs may also determine the specificity of the epigenetic response. For example, loss of methylation at the *AHRR* cg05575921 in umbilical cord blood-derived DNA was first reported to be associated with maternal cigarette smoking [106, 111]. Subsequent analyses of this region using pyrosequencing not only confirmed these findings [112], but also showed that hypomethylation of this region was no longer present in peripheral blood-derived DNA measured from the same children at 7–12 years of age [113]. These data are consistent with the malleable nature of methylation at the CG dinucleotide near the *AHRR* gene allowing it to serve as a classic biosensor, with methylation marks that are restored following cessation of the exposure.

Conversely, the DMR controlling the imprinted expression of insulin-like growth factor 2, which encodes a potent mitogenic growth factor, was reported to be hypomethylated among individuals prenatally exposed to the Dutch famine 60 years earlier as compared to their same-sex siblings [114], suggesting stability of methylation marks in this region. Prior data had shown that similar patterns of hypomethylation at this region were associated with an elevated risk of developing colon cancer [115]. In following otherwise healthy population controls from the colon cancer case–control study, these investigators also showed that methylation patterns at this region remained stable after 3 years of observation [116]. Similarly in umbilical cord blood DNA, higher methylation of the sequence region regulating the expression of *MEG3* has been associated with prenatal cadmium exposure [18], as well as neighborhood deprivation during gestation [117], described earlier. The likelihood of longitudinal stability of altered *MEG3* methylation is supported by multiple reports showing similar patterns of altered methylation in peripheral blood- and liver tissue-derived DNA of individuals with hepatocellular carcinoma as compared to controls [118–120]. Data from developmental windows such as adolescence and young adulthood are also emerging. In these studies, altered methylation at a handful of genes has been associated with air pollution exposure, of which lead and cadmium are constituents [121, 122]. Moreover, from WGBS and cadmium discussed earlier in mothers and their newborns [35] only 98 DMRs were shared between developmental windows. Based largely on DNA methylation, these and other observations suggest that there are some stable epigenetic marks that can be acquired during different developmental windows, including prenatal period, adolescence, and early adulthood [122]. Age-dependent methylation changes, like the methylation drift that occurs naturally with aging, add to this complexity. Together, these examples illustrate the challenge of interpreting human data, often from cross-sectional or case–control study designs, when the long-term stability of the vast number of methylation marks identified is unknown.

### Tissue specificity of epigenetic marks

Differential methylation that is detected in accessible tissues, such as mixed leukocytes from peripheral blood or saliva, may not always correlate with that found in the tissues/organs for which exposed-nonexposed or case–control differences are being sought. Due to the challenge of identifying exposure-induced changes in otherwise healthy tissues/cells contributing to common chronic diseases in humans (e.g. liver, brain, etc.), much of the work in this area has come from toxicology studies, among these, the Toxicant Exposures and Responses by Genomic and Epigenomic Regulators of Transcription (TaRGET) II Consortium. Initiated by the National Institute of Environmental Health Sciences, this consortium of more than 10 universities used various model systems to assess the utility of surrogate/accessible tissue epigenomic analysis to examine the role of environmental exposures on epigenomic marks in target tissues, facilitating the interpretation of human population-based studies.

Using emerging technologies, the consortium analyzed epigenetic changes caused by a wide range of environmental exposures, including toxic metals, air pollution, tobacco smoke, and endocrine-disrupting chemicals, such as bisphenol A, tributyltin, pesticides, and phthalates, in model systems that included animals, cell culture, human tissue samples, and population-based samples. The TaRGET II Consortium demonstrated that periconceptional and prenatal exposure to these environmental contaminants, caused epigenetic changes—of which the most studied were chromatin accessibility and DNA methylation, with consequent changes in gene expression [123]. Specifically, large scale DNA methylation changes were reported in blood mixed leukocytes, sustained at minimum, into young adulthood, providing distinctive molecular “fingerprints” for each exposure. Factors such as the developmental window when exposure was experienced significantly affected the nature and persistence of a wide range of epigenetic alterations. Despite the stability seen in blood, there were no substantial changes in global liver DNA methylation associated with exposures, raising questions about the suitability of peripheral blood as a surrogate for effects on target organs, and for detecting mechanisms underlying disease phenotypes [124]. Although these studies were conducted in the absence of overt phenotypic outcomes, the robust, exposure-specific epigenetic signatures identified may provide a framework for case–control studies in human populations to assess the contribution of environmental contaminants to common non-communicable diseases. Collectively, these findings lend some support that some instances, non-invasive blood-based epigenetic profiles can be used to retrospectively identify early-life environmental exposures, even when target-organ effects are subtle or not readily detectable.

Incorporating these variables is crucial for interpreting how environmental exposures contribute to disease development over the life course. Recent data from liver cancer studies demonstrate that cellular components released from the liver into circulation bear surface markers of hepatocytes, and corresponding circulating cell-free DNA has been used to measure and compare the DNA methylation of hepatocellular carcinoma relative to controls, with promising *cis*-acting DNA methylation marks identified [125, 126]. Similar progress is being made in Alzheimer’s disease, where DNA methylation changes detected from cell-free circulating DNA are being assessed as a surrogate for diseased brain tissues/cells to identify biomarkers for early detection and intervention [127, 128]. Thus, while epigenetic dysregulation due to environmental exposure can be detected in peripheral blood samples as a proxy, even within a few hours of the exposure (e.g. anesthesia administered at delivery), establishing the relationship between exposure-driven epigenetic changes in the relevant tissues to clinical outcomes, with prospects for novel diagnostic and therapeutic candidates, remains a work in progress.

### Expanding diversity in environmental epigenetics

Environmental epigenetics population studies are overwhelmingly conducted among individuals of European ancestry primarily of urban residence, and thus findings may not always be generalizable to other racial and ethnic groups or residents. Notably, summary tools such as epigenetic clocks were primarily developed with minimal inclusion of non-White populations [9]. Yet, race and ethnicity as well as rural residence, are socially constructed, context- and time-dependent classifications rather than biological variables, although they can correlate with genetic ancestry due to geographic origins, and thus, capture some genetic variation [129]. Our inability to replicate findings could in part stem from this lack of diversity [47]. The socially patterned lived experiences of racism, discrimination, and environmental exposures and unequal access to resources should be interrogated as the primary drivers of such differences. Prioritizing diverse populations requires careful considerations, as analyses that are not grounded in the social processes that produce racialized differences risk reifying biological determinism. Increasing diversity in environmental epigenetics studies is necessary but must be paired with conceptual clarity as genetic ancestry may capture population structure, whereas race reflects historically rooted and socially patterned social inequities.

### Mechanisms

Toxicology has provided much of the mechanistic insight linking exposure to environmental contaminants and pharmaceuticals with common non-communicable diseases throughout the life course, including oxidative stress and systemic inflammation. Yet, the epidemiologic and clinical literature has failed to keep pace. For example, it has been known for some time that toxic metals, such as lead and/or cadmium, as well as social stressors, are potent inducers of oxidative stress, summarized as the systemic imbalance between antioxidant defenses when compared to the production of reactive oxygen species (ROS) [130]. These metals disrupt mitochondrial function, leading to excessive ROS that can damage DNA, proteins, and lipids, contributing to cellular dysfunction and the pathogenesis of diseases like cardiovascular disease, cancer, and neurodegeneration later in life [130]. Supporting this, social stressors, including adverse childhood experiences and depression, have been associated with higher F2-isoprostane levels during gestation, increasing the risk of lower birth weight and a shorter gestational period [131]. These are established risk factors for metabolic, neurological, and cardiovascular impairment later in life. Additionally, chronic exposure to these environmental exposures triggers systemic inflammation [132], a known contributor to the initiation or progression of common non-communicable diseases throughout the life course.

Mechanisms of systemic inflammation involve activating immune cells and upregulating pro-inflammatory cytokines, such as interleukin-6 (IL-6) and tumor necrosis factor-alpha (TNF-α), creating a chronic low-grade inflammatory state [132]. Thus, oxidative stress can exacerbate inflammation, and both can drive epigenetic reprogramming, creating a vicious cycle that promotes and sustains tissue damage—increasing the risk of cardiovascular disease, diabetes, and metabolic dysfunction later in life. What remains unclear is the specificity of the epigenetic alterations across human populations. Further, we do not yet understand the potential for reversibility of these changes, whether such reversal can come from exposure avoidance, dietary modification, or therapeutic intervention. It is also unclear whether such interventions can completely restore the epigenome to its original state, or if some changes remain permanent and continue to influence disease risk. Moreover, while there is evidence that environmental contaminants like lead and cadmium can cause epigenetic alterations, it remains unclear how long these changes persist, and which marks if any can be transmitted across generations. More research is needed to better understand these mechanisms and provide a foundation for future research and policy guidelines aimed at reducing the burden of chronic diseases associated with environmental exposures.

## Strategies to address these challenges

We have the potential to address at least some of the immediate challenges faced by the field of environmental epigenetics discussed herein.

### Overcoming technological challenges

Two major technological limitations remain: the high cost of whole-genome sequencing approaches, including sequencing, data storage, and analysis at epidemiologic scale, and the lack of standardized analytical pipelines necessary to ensure reproducibility to support causal inference.

#### Cost

In the field of genetics, over 300 million single nucleotide polymorphisms (SNPs) have been identified in the human genome, including rare and population-specific variants that are now catalogued through the use of cost-effective array platforms and through imputation of haplotypes. In the epigenetics field, advances in array technology have enabled the deciphering of the methylation status of an ever-increasing number of CpG sites to address fundamental biological questions. However, unlike the cataloguing of a (usually) binary variable result for each SNP at each position, DNA methylation status is a continuous variable, thus requiring recognition of the base modification and discrimination from the non-modified base as well as quantifying the level of the modified base relative to the unmodified base for each CpG site.

With approximately 28 million CpG sites in the human genome, each of which can be measured and potentially used as a biomarker, multiple companies, led by Illumina, Inc. (with more than 930 000 CpG sites in their latest Infinium MethylationEPIC v2.0 BeadChip) and by Twist Biosciences (with up to 4 million CpG sites on their TWIST Human Methylome Panel) (TWIST Biosciences, San Francisco, CA) have developed targeted platforms that provide coverage for CpG islands (including shores and shelves), promoter regions, intragenic regions, open sea regions, and overrepresent CpG sites in genomic regions of known or suspected functions. While these arrays cover relatively small portions of the entirety of the methylome, the high throughput nature of these methods makes them cost- and time-efficient compared to whole genome sequencing.

Whole genome bisulfite sequencing—from which additional information can be gained—costs have dramatically declined over time, from upwards of $10 000 per sample in the early 2000s to as low as $200–$1000 per sample, based on specifications for the Illumina NovaSeq X, which can multiplex 64 human samples with ∼30X genome coverage. Similarly, costs for RRBS have declined from an initial ∼$5000 to ∼$250 per sample, the latter being the same approximate cost as running the Illumina BeadChip arrays. Calculated as cost per CpG, these current costs amount to roughly 2¢, 5¢, and 27¢ per 1000 CpGs by WGBS, RRBS, and Illumina BeadChip, respectively. Part of the expense for these methods is the additional manipulation required for bisulfite modification. Newer methodologies that do not require this additional chemical modification, using technology that also distinguishes unmodified cytosine from methylated and hydroxymethylated cytosine can be leveraged. These technologies also remove the biases inherent to bisulfite-based methods in that they eliminate concerns over conversion efficiency and maintain the complexity of the resulting sequence for more effective annotation back to the genome for data analysis. However, reproducibility for large epidemiologic studies remains a major challenge. Nonetheless, as these methodologies improve and mature, additional cost savings are anticipated.

#### Data analysis

Once large-scale methylation data are generated, a major bottleneck continues to be the analysis, as many environmental epigenetics researchers must seek computational resources and expertise outside their environment for storage and analysis. Cloud-based storage and computational platforms (e.g. Google Cloud Platform, Amazon Web Services, Terra, DNAnexus, etc.) are widely available and offer robust security for sensitive data, but their costs are prohibitive, limiting the volume and duration of data storage—particularly when high-dimensional methylation data are paired with exposome or poly-exposure datasets including mass spectrometry-generated data. While high-performance computing clusters available at select institutions can support these analyses, much of the generated high-throughput data remains underutilized beyond addressing primary study aims. To alleviate this bottleneck, developers should integrate existing data compression, format optimization, and data-reduction strategies—or develop new ones—within automated, reproducible workflows supported by user-friendly interfaces that reduce storage burden while preserving analytic utility.

For analysis, such data platforms could also incorporate pipelines reading in the data and sample provenance using meta data tracking. Some of this work is underway, as multiple R packages have been uploaded to Github such as BoostMe and MethImpute for whole genome methylation sequencing data, as well as iNETgrate [133, 134], to integrate DNA methylation and gene expression. These high-dimensional data also pose substantial statistical challenges—including multicollinearity, a heavy multiple-testing burden, and penalization constraints that can yield overly sparse signals. Addressing these issues requires specialized analytical frameworks such as emerging machine-learning approaches tailored for correlated, high-dimensional data, in addition to standard methods such as penalized regression, Bayesian kernel and hierarchical mixture models, and dimension-reduction methods (e.g. sparse principal components analysis, weighted quantile sum-based extensions). Implementing these advanced methods will require creating training pipelines of bioinformatics and statistical personnel.

### Overcoming tissue specificity of DNA methylation and low genome coverage

There have been multiple efforts, including the NIH Roadmap Epigenome, TARGET II consortium, and others [123, 135–138] to document methylation similarities and differences across tissues, with a particular focus on examining how internal organ and tissue methylation profiles relate to those tissues that could be used as an accessible surrogate, like peripheral blood. Findings from these efforts indicate that the tissues and cells within the body are perhaps unsurprisingly characterized by very specific profiles, with a relatively small number of genomic regions showing similar methylation profiles across tissues. Furthermore, low genome coverage in the most utilized methylation arrays, e.g. 450K/EPIC methylation arrays, has constrained environmental epigenetics research as CpG sites measurable using the arrays under-represent noncanonical regions such as enhancers or ICRs—which are highly responsive to environmental exposures. This has curtailed our ability to discover novel exposure–disease signals that may be critical for advancing mechanistic understanding and design interventions. A promising way to overcome these obstacles is the development of custom methylation profiling arrays from whole genome sequencing data. We discuss two real-world examples of such arrays, the Human Imprintome Methylation array and the Mammalian Methylation array. These platforms use the same technology as Illumina’s existing Infinium MethylationEPIC BeadChip arrays but allow for user-curated content.

#### The human imprintome methylation array

Profiling ICRs directly addresses the limitation of methylation differences based on tissue specificity and may address the challenge of methylation variation by developmental windows of exposure, providing environmental epigenetics a sharper lens to track how exposures are encoded in the epigenome, when these changes occur, and how they shape disease risk—ultimately improving early detection of chronic disease and intervention strategies. The Human Imprintome Methylation array [139, 140] is a DNA methylation profiling tool that provides dense coverage of CpG sites within known and bioinformatically predicted ICRs. This profiling tool targets DMRs that regulate the monoallelic expression of imprinted genes, which are expressed in a parent-of-origin–specific manner. ICRs are unique because their allele-specific methylation is first erased and then re-established in primordial germ cells, with allele-specificity set in the gametes—the only stage when maternal and paternal chromosomes reside in separate cells and can be differentially marked by the cellular machinery. Because methylation marks at imprinted loci are established very early in development and are transmitted stably from mature gametes through germ layer specification, tissue differentiation, and across the life course, the Human Imprintome Methylation array provides a powerful platform to investigate how environmental factors during early development might disrupt normal gene expression of a large number of genes. Approximately 1000 CpG sites from this array have now been included in the Illumina EPIC V2 bead array.

A major advantage of the Human Imprintome Methylation array is that the expected methylation pattern at profiled ICRs is well-defined and detectable across tissues. If an exposure that occurs prior to germ layer specification disrupts the ability to protect ICRs from post-fertilization reprogramming, then this “archived” change may be detectable in any tissue in the body—even decades later [114]. Moreover, because imprinted gene expression and age-related CpG sites are sensitive to specific developmental windows (e.g. prenatal, early childhood), arrays can help determine when environmental exposures archive the strongest marks. Many known imprinted genes are involved in the regulation of growth during development, including the utilization or distribution of energy (caloric usage) [141–145], and thus are logical targets for studying the early origins of human adult-onset diseases, in addition to their dysregulation in cancers, using accessible cell types obtained at later ages. These unique features of ICRs, namely, their early establishment, longitudinal stability, known locations, and similarity across cell and tissue types, can facilitate their use as archives of early developmental exposure(s) or predictors of future disease risk, helping link the environment, the epigenome, and health outcomes, in accessible tissues.

#### Mammalian methylation arrays

The mammalian methylation array [146] is a powerful custom DNA methylation profiling platform designed to measure methylation at highly conserved CpG sites across multiple mammalian species, thereby facilitating cross-species comparisons and advancing the translation of environmental epigenetics findings. With these arrays specifically designed for model organisms like mice, rats, or primates, environmental epigenetics utilizes these models to gain insights into epigenetic responses to environmental exposures in humans. Mammalian arrays have improved our understanding of human epigenetics by enabling large-scale, comparative studies across tissues, developmental stages, and species. Researchers can use these arrays to study a wide range of exposures [147]. Such cross-species comparisons can reveal causal mechanistic links for how early-life exposures can have long-term health effects, supporting the developmental origins of health and disease hypothesis [148]. These arrays also can allow more accurate construction of DNA methylation “clocks” across species [149], deepening understanding of biological aging and its links to disease. These arrays have the potential to contribute to our understanding of subclinical disease progression and are being explored for early detection and prognostic biomarkers, and targets for possible therapeutic intervention.

Ongoing efforts are underway to develop custom methylation arrays for stochastically established DNA methylation marks in correlated regions of systemic interindividual variation (CoRSIVs) [150, 151].

## Summary and conclusion

While the nascent field of environmental epigenomics continues to grow to improve our understanding of the effects of the wider exposome, building a compendium of epigenetic marks responsive to environmental exposures requires overcoming existing limitations. Custom DNA methylation array examples illustrate how specific features of the genome, e.g. stability of DNA methylation marks over time, established function, and similarity of methylation marks across species, could be harnessed for other developmental windows shown to influence adult-onset common non-communicable disease. Overall, custom methylation arrays provide a powerful and scalable tool to investigate the plasticity of the epigenome and response to environmental exposures and uncover epigenetic biomarkers of exposure and perhaps disease. These arrays can also offer insights into the mechanisms by which epigenetic regulation influences health across the lifespan, overcoming tissue specificity. For example, infancy, early childhood, and puberty are vulnerable developmental windows during which exposures to a wide range of environmental exposures have been linked to increased risk of multiple cancers, cardiovascular and metabolic diseases, and neurological disorders, albeit inconsistently. Tissue specificity at these latter windows can be addressed by harnessing the growing area of cell-type deconvolution to control for cellular heterogeneity within the surrogate samples analyzed, and to identify individual surrogate cell type methylation profiles that are normally correlated with those in the relevant but inaccessible target cells or tissues in healthy individuals, but diverge in the affected individuals [152, 153]. Using whole genome approaches to develop such methylation arrays for a set of developmental windows, while accounting for cell types contributing the DNA assayed for CpG methylation, can provide a handful of platforms in which exposure-related epigenetic data can be linked to past exposures and with that, help predict, prevent, and develop intervention strategies to reduce future risk common non-communicable diseases.

## Data Availability

The original contributions presented in the study are included in the article/Supplementary material, further inquiries can be directed to the corresponding author/s.
